# “It Just Rips My Heart Out”: Child Custody Dispositions After Women Leave Abusive Partners

**DOI:** 10.1177/10778012251329259

**Published:** 2025-03-25

**Authors:** Leslie M. Tutty, H. Lorraine Radtke, Kendra L. Nixon

**Affiliations:** 1 University of Calgary, Calgary, AB, Canada; 2 University of Manitoba, Winnipeg, MB, Canada

**Keywords:** intimate partner violence, child custody, violence against women, family court

## Abstract

Family court child custody dispositions for women who have left violent partners can result in complications and difficulties. This secondary analysis of data from 2005 to 2009 describes the child custody dispositions of 369 Canadian women survivors of intimate partner violence (IPV) (48.5% Indigenous, 44.7% White, and 6.4% Visible Minority). Of these, the most common court disposition was mothers receiving sole custody (38.9%), then those who did not use formal custody (34.5%), joint custody (13.3%), fathers receiving sole custody (4.4%), and family court still in progress (8.8%). Mothers’ perspectives about these dispositions were qualitatively analyzed. Implications for researchers and practitioners are presented.

Acknowledging that many divorced fathers desire to be active participants in their children's lives and that contact with their fathers may be in the children's best interests, most US and Canadian family courts demonstrate a preference for allowing maximum child access to both parents, either through joint custody or generous child visitation (Brinig et al., 2014; Godbout et al., 2015). However, whether such access is the best option for divorcing couples in which the mother has been abused by the father has been debated for decades (Bancroft & Silverman, 2000; Brinig et al., 2014; Drozd et al., 2004). Further, we know relatively little about the child custody arrangements of women who have left abusive partners, the focus of the current study.

## Intimate Partner Violence

Violence against women is a significant global problem that results in injury, emotional harm, and, at worst, death (Dawson et al., 2009; World Health Organization, 2021). The abuse that women endure from intimate male partners takes many forms and most often extends throughout the relationship. Intimate partner violence (IPV) is different from the marital disagreements that all couples experience. Heterosexual intimate partner abuse against women is not about anger in reaction to disputes but the intentional and instrumental use of power to control the woman's actions (Kimmel, 2002), appropriately termed “coercive control” (Stark, 2007).

Most people assume that leaving an abusive partner ends the violence. However, IPV commonly continues after women leave (DeKeseredy et al., 2004; Hardesty & Chung, 2006) either through continued physical assaults (Brownridge et al., 2008; Rezey, 2020), sexual assaults (DeKeseredy et al., 2004), or coercive controlling tactics (Hay et al., 2023; Hayes, 2012; Tutty et al., 2024). When the couple share children, visitation provides the opportunity for the IPV to continue (Feresin et al., 2019; Morrison, 2015; Toews & Bermea, 2017; Tutty et al., 2025).

## Child Custody in the Context of Intimate Partner Violence

When women separate from abusive partners, a bitter, high-conflict divorce often results, although Archer-Kuhn (2018) warns that domestic violence and high-conflict divorces are not the same conceptually, with coercive control a key aspect of the former. When formal legal channels are followed to end a relationship, mothers must navigate a variety of policies and procedures related to property, financial support, and child custody, with custody arrangements playing a critical role in shaping the nature of the contact between the women and their abusive partners (Shepard & Hagemeister, 2013). Moreover, the occurrence of IPV is a common characteristic of high-conflict cases: Morrill et al. (2005) estimated that in the United States, as many as 50% of disputed child custody cases involve IPV. Custody battles are frustrating and stressful especially in the context of IPV according to Shalansky et al. (1999), who noted that many women perceive their partners as using the legal system to continue controlling them and believe that the courts pay insufficient attention to the abuse. Family court judges have consistently been accused of not acknowledging or understanding the consequences of male-perpetrated IPV (Jeffries, 2016; Morton et al., 2021; Rosnes, 1997).

The importance of maintaining the father–child relationship is a key belief of most family law practitioners (Lessard et al., 2010). Across studies, even when evidence of a perpetrator's intimate violence exists in the criminal justice system, family courts often do not know of this history (only 25% were aware of the IPV in family court in Kernic et al., 2005), ignore or dispute the evidence (Busch & Robertson, 2000; Gutowski & Goodman, 2019; Khaw et al., 2021; Miller & Manzer, 2021; Saunders & Oglesby, 2016; Silverman et al., 2004), or do not believe the mother's claims (Bemiller, 2008; Saunders et al., 2016). Importantly, even when the family courts had substantiated information about an abuser's history, many still allowed fathers access to their children, often unsupervised (83% in Kernic et al., 2005; 46% in Silverman et al., 2004). In the Canadian context, abusive men are largely awarded unsupervised access to their children (Shaffer & Bala, 2003).

The quality of parenting by fathers who have abused the children's mother (and sometimes the children) may not justify the access accorded to them by the legal system. Several researchers have raised concerns over the quality of fathering from at least some men who use IPV (i.e., Bancroft & Silverman, 2000; Cater & Forssell, 2014; Humphreys et al., 2019), citing under-involvement and seldom taking parental responsibility. Thompson-Walsh et al.'s (2018) qualitative study of 20 fathers, half of whom had abused their partners, concluded that the abusive men generally perceived their ex-partners as bad mothers and blamed them for the difficulties co-parenting.

Consistent with this, mothers and children who had experienced IPV in the United Kingdom reported that fathers/perpetrators had a substantive negative impact on both mothers and children, thereby jeopardizing the known benefits of a positive mother–child relationship (Katz et al., 2020). This analysis of 29 children's perceptions of their father's use of coercive controlling tactics toward the children post-separation, showed three disturbing patterns. While the fathers who had perpetrated IPV often appeared “dangerous” and made the children feel unsafe, in more extreme cases they appeared “omnipresent,” resulting in the children being constantly fearful and vigilant. A third pattern, described as “admirable,” entailed the father “playing the role of a caring, indulgent, concerned and/or vulnerable-victim father” (p. 317). Such fathering, when combined with dangerous fathering, served to confuse and manipulate children who were both drawn to the prospect of a loving father but then simultaneously fearful of him. Playing the victim role with the children and in public contexts with professionals such as teachers, police, and social workers served to mask the abuse and cast suspicion on the mother. Thus, fathers’ use of coercive control with their children is a potential danger to be considered when custody decisions are being made in the context of IPV. Altogether, this research suggests caution in assuming that children need their fathers to be a part of their lives.

Concerns about how fathers’ coercive control impacts decision-making on child custody are long-standing. For example, in their study with 24 Canadian women, Varcoe and Irwin (2004) concluded that child custody and access processes provided opportunities for abusive partners to exert control over their ex-partners. Reflecting the growing empirical evidence of the frequent continued use of coercive control tactics in legal arenas by authors such as Archer-Kuhn (2018), Douglas (2018), and Gutowski and Goodman (2023), Elizabeth (2017) documented a subset of legal abuse specific to custody issues that she terms “custody abuse.” Rivera et al. (2012) described these child custody actions as “procedural abuse” (also called legal abuse or litigation abuse by authors such as Gutowski & Goodman, 2023), providing examples such as the ex-partner “tried to get/received custody or visitation to stay in your life,” “tried to get/received custody or visitation to get back at you,” and “threaten to take you back to court for support, custody, or visitation.” (p. 2786). One of the most damaging is accusing partners (typically mothers) of “parental alienation” (Jaffe et al., 2008a; Meier, 2009; Sheehy & Boyd, 2020; Van Horn & Groves, 2006).

Child protection services may contribute to problematic decision-making regarding child custody post-separation through failure to recognize the serious implications of IPV. In a recent Ontario study of child welfare investigations (Black et al., 2021), about one-third of referrals involving a child custody dispute were made by the custodial parent, entailed allegations of exposure to IPV, and most were deemed to be nonmalicious referrals. Nonetheless, child protection cases involving custody disputes were less likely to be kept open and followed up by child welfare services than those not involving a custody dispute. Given existing research (i.e., Tutty et al., 2024), it is highly likely that many of the cases where IPV had been reported entailed coercive control tactics exercised by the father and, thus, these results are troubling. Black et al. argue that child protection professionals may lack knowledge about IPV and how to appropriately manage the complexity of cases where IPV and custody disputes coincide.

The judicial system within which custody decisions are made is another important area where concerns about limited knowledge of IPV have been expressed. Some have questioned whether family courts understand the nature of IPV, preferring to ignore the gendered nature of IPV against women or its effects on children (Morton et al., 2021). In response to such concerns integrated or unified family courts in which criminal and custody issues are dealt with in one courtroom (Schwarz, 2004) with the intention of affording full knowledge of the case to lawyers and judges have been implemented in some jurisdictions in Ontario (Birnbaum et al., 2017; Steinberg, 1999) but not across Canada (Jaffe et al., 2008b). Empirical research exploring the experiences of women navigating the system that determines child custody arrangements post-separation in the context of IPV may offer additional persuasive arguments for the need to further transform the judicial system.

## Rationale for the Current Study

Studies of women's perspectives about custody and access issues are not common. Most are qualitative, with samples from 12 to 39 (Bemiller, 2008; Elizabeth, 2019; Gutowski & Goodman, 2019; Hughes & Chau, 2012; Khaw et al., 2021; Miller & Manzer, 2021; Shepard & Hagemeister, 2013), three of which focused on women who had lost custody (Bemiller, 2008; Elizabeth, 2019; Khaw et al., 2021). While qualitative studies provide important details about the experiences of a limited number of respondents, only a few have used quantitative methods (Birnbaum et al., 2017; Logan et al., 2003; Morrill et al., 2005). Two of these studies identified a strong tendency to award joint custody even in the context of IPV. Logan et al.'s (2003) study of a random sample of 66 divorcing couples with children in Kentucky, of which 33 had experienced IPV, found no significant differences in the court dispositions between the IPV and the non-IPV groups”: 41% of the IPV cases were awarded joint custody with the mother as a primary residential parent, 37% were awarded sole custody to the mother; 14% sole custody to the father and 6% split residential custody. Morrill et al. (2005) examined the experiences of 393 abused women across six US states, finding that 40% of the abusive fathers were awarded joint custody. Ogolsky et al. (2023) uniquely matched 190 US mother's self-reports of IPV and their actual court documents (i.e., divorce, civil protective order, and criminal court record), concluding that IPV was generally not documented in divorce cases.

More recently in Canada, however, Birnbaum et al. (2017) found a different pattern when comparing dispositions made in child custody cases with at least one domestic violence (DV) charge in an integrated family/domestic violence court (IDVC) that entailed interventions aimed at reducing violence with those made in a nonspecialized family court (the comparison group). Of the final child custody dispositions in the comparison group (*N *= 160) (at least one DV charge, no integrated DV family court) 84.8% of the mothers were awarded custody compared to 66.7% of the mothers in the IDVC group. Joint decision-making with primary residence being with the mother and shared custody was awarded in 7.6% to the father, 6.9% joint decision-making with the mother having primary custody, and 0.7% shared custody in the comparison group compared to 15.5% of cases in the IDVC group.

Although this has been a topic of interest to several researchers, more can be learned about best practices in making custody decisions in the context of IPV. Additional studies examining the experiences of diverse Canadian women from ethnic/racial groups and with varied life circumstances would be helpful. To fill that gap, the current study utilizes data from “The Healing Journey,” a longitudinal study of women with a history of intimate partner abuse across the Canadian prairie provinces. Our analysis explores the custody arrangements of 369 women using quantitative methods but also includes qualitative analysis of their perceptions of those arrangements, including the impact on themselves and their children. We addressed three research questions: (1) Did family court personnel know about the IPV? (2) Do mothers with different custody dispositions differ in terms of ethnic/racial identity? and (3) Do fathers with different custody dispositions differ in terms of ethnic/racial identity? As noted above, the last two questions address a gap in the literature that, were differences in racial/ethnic identity noted, could have clear implications for everyone involved, including mothers and court personnel.

Importantly, almost half of the women identified as Indigenous, a group that is at higher risk for IPV (Brownridge, 2008: Heidinger, 2022) and for post-separation IPV (Pederson et al., 2013). Indigenous children are more likely to have been maltreated (Brownridge et al., 2017), a factor that must be considered in child custody cases. Both IPV and child abuse are linked to the historical trauma from the colonialism of Indigenous peoples (Brownridge et al., 2017; Daoud et al., 2013; Ogden & Tutty, 2023). Further, Indigenous women often have negative experiences with the criminal justice and legal system (Jock et al., 2022), and we know little about their child custody after IPV.

## Methodology

This exploratory secondary analysis used a quantitative approach with qualitative comments from several open-ended questions (Sandelowski, 2000a). The data were from the “The Healing Journey,” a longitudinal, Canadian study with a convenience sample of 665 women who had experienced IPV in the three prairie provinces of Alberta, Saskatchewan, and Manitoba. The original study assessed the impact of IPV on women, including mental health and general well-being (Tutty et al., 2021a), experiences of mothering (Ateah et al., 2019; Nixon et al., 2017), and how they fared over the course of 2.5 years (Tutty et al., 2021b). Both academics and community agency research team members designed the research, recruited participants, and interpreted the results.

Data were collected in seven waves between 2005 and 2009, with one wave specific to an economic analysis (DeRiviere, 2014). The survey instruments included questions with specific response options (e.g., “yes/no” or multiple choice) that made them amenable to quantitative analysis and open-ended questions appropriate for qualitative analysis. Trained interviewers read the questions to the participants and recorded their responses.

The research protocols were approved by the Ethical Review Boards of the six associated universities (Universities of Calgary; Manitoba, Regina, Saskatchewan, Brandon & Lethbridge). The women were recruited through posters from VAW shelters or counseling agencies. The criteria for inclusion were a minimum of 18 years of age; the most recent incident of IPV no sooner than 3 months and no longer than 5 years prior to participation in the study; commitment to stay in the study for the full 2.5 years; and no debilitating mental health issues. Honoraria of $50 CAN were given to participants at each wave.

### Quantitative Data Analysis

The current study used quantitative analysis to describe the demographic characteristics of the women, including their custody arrangements, and the extent of the IPV experienced. IPV was measured by The Composite Abuse Scale (CAS) (Hegarty et al., 2005), a 30-item self-report questionnaire with four subscales: Severe Combined Abuse, Emotional Abuse, Physical Abuse, and Harassment as well as a Total CAS score. The measure asks whether partners took certain actions (in the past 12 months) and the frequency of such actions on a six-point Likert scale of never (0), only once (1), several times (2), once per month (3), once per week (4), or daily (5). for a total score of 150 (Hegarty et al., 2005). The clinical cut-off for the entire scale is 3 to 7 (Hegarty et al., 2005). The CAS has strong criterion and construct validity, as well as internal reliability (*α* = 0.85); the subscales also have a Cronbach's alpha of 0.85 or above (Hegarty et al., 2005). Cronbach's alpha in the original Healing Journey study is .93.

Pearson's chi-square analysis, with effect sizes calculated with Phi or Cramer's *V*, was used to compare demographics with respect to the different forms of custody. Standardized residuals identified category differences responsible for the statistically significant chi-square analysis (Field, 2009). Effect sizes were interpreted using Rea and Parker's (1992) suggested benchmarks of under .10 as a “negligible” association; between .10 and under .20 as “weak”; between .20 and under .40 as “moderate”; and between .40 and under .60 as a relatively “strong” association (p. 203).

### Qualitative Data Analysis

Descriptive qualitative analysis (Neergaard et al., 2009; Sandelowski, 2000b) was used to examine comments about the custody experiences of 369 mothers whose intimate partners had abused them and who noted that the custody of their children was or had been an issue, whether family court personnel knew of the IPV and the effects of the custody arrangements on the woman and her children. This approach to qualitative analysis is particularly appropriate for mixed-methods research (Neergaard et al., 2009) and for “assessing, developing and refining interventions with vulnerable populations” (Sullivan-Bolyai et al., 2005, p. 127).

The analysis of the responses to the open-ended questions followed established descriptive qualitative analysis processes (Sandelowski, 2000a): We identified the major themes (Graneheim & Lundman, 2004; Neergaard et al., 2009; Thorne, 2000) with respect to each type of custody arrangement since each has distinct characteristics. First-level coding entailed word-by-word scrutiny of the comments to identify prominent themes and subthemes (Braun & Clarke, 2006). Second-level coding involved looking within the themes and subthemes to identify similarities, differences, and gaps using the constant comparative method (Thorne, 2000).

Some typical mechanisms to establish trustworthiness in qualitative research, such as member-checking, are not possible in secondary qualitative analysis (Yardley et al., 2014). However, consistent with Lincoln and Guba (1985), the quotes were triangulated by source (more than one respondent raising similar issues), analyst, and negative case analysis and this triangulation was used to establish dependability.

## Results

### Demographics of the Research Participants

The current analysis focused on 369 women (of 665 or 55.5%) who were separated from their partners in Wave 1 of the study (see Table 1). The women's average age was 34 years while their ex-partner's ages were an average of 36.4 years. Their ethnic/racial identities were 48.5% Indigenous, 44.7% White, and 6.4% Visible Minority; while the ex-partners were Indigenous (46.7%), White (45.6%), and 7.7% Visible Minority. The largest groups in the women's Visible Minority category were African Canadian (8 or 32%), Latin American (6 or 24%), and South Asian (5 or 20%). Two-thirds (66.6%) of the women were from urban locations (100,000+), with smaller numbers from rural/remote (under 29,999) (17.9%) or small towns/cities (30,000–99,999) (15.5%).

**Table 1:** Demographics of Mothers Affected by Custody (*N* = 369).

Variable	Categories	
Age in years (*N* = 367)		34 (18–55) (*SD *= 7.8)
Ex-partner age (*n* = 369)		36.5 (18–72) (*SD *= 8.8)
Total yearly income (past year) (*N* = 339)		$22,043 (0–$235 K) (*SD *= 24,636)
Ethnic identity (*n* = 367)	Indigenous	178 (48.5%)
	White	164 (44.7%)
	Visible minority	25 (6.4%)
Ex-partner ethnic identity (*n* = 364)	Indigenous	170 (46.7%)
	White	166 (45.6%)
	Visible minority	28 (7.7%)
Urban/rural (*N* = 368)	Urban (100,000+)	245 (66.6%)
	Small cities (30,000–99,999)	57 (15.5%)
	Rural/remote (less than 29,999)	66 (17.9%)
Relationship status (*N* = 368)	Separated/divorced/ex-common law	262 (71.2%)
	Married/common law	34 (9.2%)
	Boyfriend	13 (3.5%)
	Ex-boyfriend	59 (16%)
Age of oldest child (*n* = 369)	Under 18 years	307 (83.2%)
	Oldest is adult but others under 18	62 (16.8%)
Highest education (*n* = 369)	Not completed HS	140 (37.9%)
	Completed high school or GED	87 (23.6%)
	Post-secondary—technical	68 (18.4%)
	Post-secondary—university	74 (20.1%)
Currently working (*n* = 363)	Full-time	95 (26.2%)
	Part-time/casual	60 (16.5%)
	Not working	208 (57.3%)
Custody type (*N* = 368)	No formal or unclear	127 (34.5%)
	Mother has sole custody	143 (38.9%)
	Joint custody	48 (13.0%)
	Father has sole custody	19 (5.2%)
	Custody in process	31 (8.4%)
Combined abuse scores (Wave 1) (*N* = 363)	CAS severe combined abuse	7.5 (*SD *= 7.1)
	CAS emotional abuse	28.8 (*SD *= 13.9)
	CAS physical abuse	12.5 (*SD *= 8.4)
	CAS harassment	8.1 (*SD *= 5.3)
	CAS total	55.9 (*SD *= 28)

With respect to their education, 37.9% of the women had not completed high school, 23.6% had completed high school, while 38.5% had some post-secondary education, either in technical institutes (18.4%) or universities (20.1%). Their average total income in the past year was $22,043 CAN; about half of the women's incomes fell below the poverty line for that time in the three Canadian provinces (DeRiviere, 2014). This low yearly income is partly explained by the women's employment status: 57.3% were not currently working, another 16.5% worked casually or part-time and only 26.2% worked full-time. Scores on the CAS subscales and Total scores were well above the suggested clinical cut-off scores for partner abusive behaviors, indicating that the women had experienced severe and varied forms of abuse.

### Child Custody Dispositions

Of the 369 women, slightly more than one-third (127 or 34.5%) had no formal custody agreements from a family court or a disposition could not be ascertained. Of this group, the mothers retained sole custody of the children in all but three cases. In cases that were heard in family court and a disposition given (*N *= 210), the majority awarded sole custody to mothers (143 or 68.1%), another 23.3% (48 cases) were awarded joint custody, and in 19 cases (9%) sole custody was awarded to the fathers. Regardless of whether there was a formal custody arrangement established through the court system, most mothers (317 or 86.1%) had sole custody of their children, with 40 (10.9%) of fathers having sole custody. In 11 cases (3%) children spent approximately half their time with each parent.

In cases that were heard in family courts, almost two-thirds (60.6%) of the women reported that both the lawyers and judges knew of the IPV, while about one quarter (26%) indicated that only the lawyer was aware, and 13.4% declared that no one was informed (see Table 2). Awareness of IPV did not affect the type of custody awarded (*χ*^2^* *= 4.2; *p *= .65). This analysis addressed our first research question.

**Table 2:** Do Family Court Personnel Know About IPV? (*N* = 127).

	Neither judge nor lawyer knows	Lawyer knows	Judge and lawyer know	Total
Mother has sole custody	11 (14.3%)	17 (22.1%)	49 (63.6%)	77
Joint custody	4 (13.3%)	9 (30%)	17 (56.7%)	30
Father has sole custody	1 (14.3%)	1 (14.3%)	5 (71.4%)	7
Custody in process	1 (7.7%)	6 (46.2%)	6 (46.2%)	13
Total	17 (13.4%)	33 (26%)	77 (60.6%)	127

*χ*^2^* *= 4.2; *p *= .65

As can be seen in Table 3, there were significant differences in the types of custody arrangements and the use of the court system based on the ethnic/racial identities of the women. Women who identified as White were significantly more likely to use family court processes compared to women who identified as Indigenous and less likely to have an informal custody arrangement, and women who identified as Indigenous and used the formal court system were less likely to have been awarded joint custody compared to women who identified as White (*χ*^2^* *= 21.3, *p *= .006; Cramer's *V *= .16, a small effect). This analysis addressed our second research question. Further, fewer fathers with reported Indigenous identity were awarded joint custody compared to fathers with reported White identity (*χ*^2^* *= 21.2, *p *< .0; Cramer's *V *= .17, a small effect) (see Table 4). This analysis addressed our third research question.

**Table 3:** Type of Child Custody by Mother's Ethnic/Racial Identity (*N* = 366).

Custody type	White	Indigenous	Visible minority	Totals
No formal custody or custody unclear	42 (25.6%)*	75 (42.1%)	10 (41.7%)	127 (34.7%)
Mother has sole custody	63 (38.4%)	69 (38.9%)	9 (37.5%)	141(38.5%)
Joint custody	30 (18.3%)	14 (7.9%)	4 (16.7%)	48 (13.1)
Father has sole custody	10 (6.1%)	8 (4.5%)	1 (4.2%)	19 (5.2%)
Custody in process	19 (11.6%)	12 (6.7%)	0	31 (8.5%)
Totals	164	178	24	366

*Standardized residuals significant at *p* < .05.

*χ*^2^* *= 19.4, *p *< .013; Cramer's *V *= .16

**Table 4:** Type of Child Custody by Father's Ethnic/Racial Background (*N* = 363).

Custody type	White	Indigenous	Visible minority	Totals
No formal custody or custody unclear	43 (26.1%)	73 (42.9%)	9 (32.1%)	125 (34.4%)
Mother has sole custody	67 (40.6%)	62 (36.5%)	12 (42.5%)	141(38.8%)
Joint custody	33 (20%)*	12 (7.1%)*	2 (7.1%)	47 (12.9%)
Father has sole custody	7 (4.2%)	9 (5.3%)	3 (10.7%)	19 (5.2%)
Custody in process	15 (9.1%)	14 (8.2%)	2 (7.1%)	31 (8.5%)
Totals	165	170	28	363

*Standardized residuals significant at *p* < .05.

*χ*^2 ^= 21.2, *p* < .007; Cramer's *V *= .17

### Mother's Perspectives of the Custody Decisions

The mothers’ comments regarding custody arrangements were diverse and indicated both positive and negative experiences. The following sections detail their perspectives on their custody arrangements and how they have affected both the mother and her children, beginning with the least mentioned, fathers awarded child custody, mothers awarded child custody, joint custody, custody in process, and no formal custody process.

#### Father has sole custody (*n *= 22)

Although the women did not necessarily disclose reasons for the court dispositions, 22 cases (19 were family-court involved) were unique in that four women disclosed their own addiction issues, one reported mental health problems, and one identified health issues that they associated with the father being awarded custody. Comments regarding this included, “I’m pissed that the courts did not believe that there was abuse or addictions on his part; he came off looking like angel in court, while I admitted to my addictions;” “I lost custody as their father has proved that I am drinking all the time and can’t care for the children;” and “I was working three jobs. I had a nervous breakdown and gave custody to my baby's father.”

Two mothers mentioned that they had lost custody because of lack of support from court personnel: “The lawyers and judge knew about the abuse. They ended up blaming me. He had the money, background, and the right lawyer,” and “Husband and family had money and support and I didn’t. Didn't go into abuse. He tried to pay my lawyer to not show up.”

The three cases without family court dispositions were worked out privately, although in one case without the mother's agreement, “We verbally agreed he would be primary residence. I really didn’t have any say. I allowed family members on both sides to place [daughter] with her dad.” In another case, “The father of the 11-month-old co-parents with me but the baby lives with him. No court-ordered arrangements – just worked it out privately. It's better for the kids. They know what I am going through.”

As might be expected, the mothers who had lost custody of children missed their children and most were depressed or angry. Some examples include: “It was safety for son. He didn’t have to see his mom abused. I get lonely. I try to hide the abuse from him to protect him;” “Miss my children terribly. Very difficult to see my sons. They are out of province. When daughter cries because she doesn’t want to go home with father, I feel hopeless;” and “All I ever wanted was to be a Mum, and now that is taken away from me.”

In most cases, the mothers perceived their children as sad or confused and missing their mothers. For example: “Sons miss me terribly; however, they know they are loved. Daughter is stressed that she has to stay with father.” “My son has abandonment issues but when he's older I’ll try to work through that with him.” “My daughter's being ripped apart. My son's too little to know – he hasn’t even bonded with me.” In one tragic occurrence, a young preschooler with an Indigenous identity died from a physical assault when living with his father who had been granted sole custody.

#### Mother has sole custody (*n = *143)

A portion of the mothers who had been granted sole custody spontaneously remarked on the advantages of this family court disposition. Twenty-five mentioned feeling safer or more settled themselves (e.g., “I am happy, relieved;” “I like that I have sole custody. I wanted it so that he wouldn’t be able to use my kids against me;” and, “Happy I have sole custody of one boy; this gives me a second chance to be a mom.”). Twenty-one mothers expressed relief that their children were safer, commenting, for example, “I’m glad I don’t have to share my children with him because he would hurt them more;” and “I have a more secure feeling over my children.”

Sixteen mothers felt the weight of their responsibilities being a single parent (e.g., “I don’t get a break;” “It can be overwhelming financially; it is expensive to clothe and feed four small children;” “I wish they had a male figure to guide them, especially my sons. I must work extra hard to make them feel secure and confident;” and, “I get angry at my ex-partners because they take their responsibility as father and parents lightly. As a single parent I find I don’t get much time to myself. Sometimes I feel overwhelmed”).

On the other hand, sole custody represented little change to the realities of some mothers’ everyday lives (e.g., “I was primary caregiver for [the] children even when with partner”) or a significant improvement (e.g., “I feel very peaceful”). The fathers were not always as invested in their parenting responsibilities as might be considered ideal. As one mother mentioned, “I like parenting on my own, I have pride in that I am raising three wonderful children on my own.”

Several mothers mentioned their belief that it is important for children to have relationships with their fathers (e.g., “Beneficial for the kids. When I left, I didn’t plan to keep them away from him;” and, “I want my son to have a relationship with his father. There are some questions my son has that a father can address better than a mother.”).

Regarding whether the family court personnel knew about the IPV before awarding the mother sole custody, in about two-thirds of cases (63.6%) both the lawyers and judges were aware of the IPV but in several instances the lawyer knew but chose not to inform the judge, commenting: “Lawyer knew about the abuse, but did not bring it up in court, because he believed that it was too hard to prove;” “My lawyer didn't bring it up in court as his [ex-partner] lawyer was trying to prove I was mentally incapable to be a parent.”

While the custody issues seemed settled in cases where the mothers were awarded sole custody, four mentioned difficulties regarding receiving child support payments, two were concerned that their partners might abduct their children, and one mentioned her partners’ attempt to keep her from moving to another locale.

#### Joint custody (*n *= 49)

The children lived primarily with the mother in 31 of the cases with joint custody (63.3%), primarily with the father in nine cases (18.4%), and with time divided 50/50 in another nine cases (18.4%). Subthemes from the mother's comments who had sole custody were similar to the previously noted impressions of mothers with sole custody.

In five of the nine cases in which the children primarily lived with their fathers, it was the child's choice, e.g., “12-year-old son – joint custody but we have agreed that he will live with father at son's request,” and “My son (17 years) lives with father in B.C. because he wanted to. I was very sad, tried to visit him as much as possible.” Notably, most were boys, and all were 9.5 years or older. In another case, the mother was at school and anticipated becoming the primary caretaker again after graduation. In the final three cases, the mothers were not happy about their children primarily living with the fathers.

In most of the cases where custody was shared 50/50 (7 of 9), the mothers were unhappy that this was the case, but three noted that their child/ren had a say in this decision: “They don’t complain about going back and forth. They are old enough to choose or stop but they choose to keep going to their father's house;” “We share joint custody 50/50. My daughter stays with me for two nights, then for two nights with her father. I am heartbroken. I would rather have her more of the time, but this is what she wanted, so I have to accept it;” and “He goes back and forth between us. Crushes me that he wants him all the time. When my son is with me, he is watching everything because he is to report back to his dad.”

Three mothers, all having been awarded joint custody and having arranged a 50/50 split with the fathers, described on-going custody battles. These two examples suggest the father's use of the courts as a tactic of coercive control:

He gets my daughter half the time. We were just in court yesterday and he's now arguing for primary care and control, but I know he isn’t capable or willing to take on that role and responsibility. He's doing it to cause me problems. He keeps bringing up me working as a reason for the courts to award him custody. It is very difficult for her; he puts words in her mouth and sticks her in the middle of his disdain for me.

We were sharing custody informally then he got mad and decided to use parental alienation. Now 1/2 – 1/2 for younger son. Ex told me I would lose kids forever if I didn't comply with what he wanted. When the kids live with him, they don’t do well. They are recognizing their Dad's behavior for what it is but try to keep the peace by living with dad as much as he wants. They get caught in the middle.

#### Custody cases in progress (*n *= 31)

Of the cases that were still in process in family court, most of the children resided with their mothers (26 or 83.9%), while four lived mostly with their fathers (4 or 12.9%). In a final case, the children spent relatively equal time with both parents (3.2%). Quotes from the mothers with custody in progress were similar to the comments from those in each of the sections: mothers with sole custody, fathers with sole custody and joint custody.

#### No formal custody or custody status unclear (*n *= 127)

In a little more than one-third of the cases (127 or 34.7%), there was no formal arrangement for child custody, or the women did not specify any family court decision. In the majority (116 or 91.3%), the children lived with their mothers. In the 11 cases where children lived with their fathers, four mothers accepted this arrangement and one did not, as she had had no input into the arrangement. In the other six cases, the arrangement worked out well for the mother, e.g., “It has given me the freedom to work. But it has also given me time with the addictions,” “Four kids – mutually agreed to take care of kids while I get back on my feet;” or was better for the children, “I have left my son with his dad until he completes his schooling; later he will join me.” “It's a big decision but it's better for the kids – they know what I am going through.” “I have one bedroom. Not enough for four teens. I’m not happy but can’t change this.”

## Discussion

Although the original data was collected in 2005–2009, this study afforded a unique opportunity to examine the child custody dispositions of a large sample of women abused by intimate partners and now separated from abusive partners, including a substantial number who did not use the family court system. Notably, though, the data was collected long before recent changes to Canada's Divorce Act (Government of Canada, 2024) that attempts to include considerations about the impact of IPV in family court cases. In earlier qualitative research on family court dispositions (Bemiller, 2008; Elizabeth, 2019; Gutowski & Goodman, 2019; Hughes & Chau, 2012; Khaw et al., 2021; Miller & Manzer, 2021; Shepard & Hagemeister, 2013), all of which had small samples of from 12 to 39 women, three focused solely on mothers who had lost custody (Bemiller, 2008; Elizabeth, 2019; Khaw et al., 2021).

The three research questions addressed in the quantitative analysis focused on the experiences of mothers who used the formal justice system to determine child custody arrangements post-separation. Our analyses indicated that in most of such cases, both the lawyers and the judges were aware of the IPV but, in almost 40% of cases, either only the lawyer was aware, or neither lawyers nor judges knew of the IPV (research question 1). This is consistent with previous literature documenting mothers’ reluctance to disclose IPV for fear of receiving negatively biased treatment within the family court system and lawyers’ withholding the information for similar reasons (e.g., Bemiller, 2008; Gutowski & Goodman, 2019; Miller & Manzer, 2021; Ogolsky et al., 2023). Women's fears of their abusive partners using the family court system to continue a pattern of coercive control post-separation (i.e., legal or litigation abuse) is also well-recognized and another reason women may remain silent about the abuse for fear of provoking retaliation (e.g., Archer-Kuhn, 2018; Elizabeth, 2017; Hay et al., 2023; Lux & Gill, 2021; Tutty et al., 2024).

There was no relationship between court officials' awareness of the IPV and the type of custody awarded, which appears problematic. Although, in most cases (68.1%), custody was awarded to the mother, a sizable minority that were awarded joint custody or sole custody to the father (31.8%) involved mothers who had experienced severe forms of IPV. Education for the judiciary is essential, a prime example being “Keira's Law” (Mundie, 2023), a recently adopted Canadian law designed to provide critical information about coercive control and other facets of IPV. Making the court system more welcoming to women and responsive to IPV and its consequences for women and children remains a priority (e.g., Lux & Gill, 2021; Ogolsky et al., 2023).

We also assessed the relationships between custody decisions and the ethnic/racial identities of the mothers and the fathers (Questions 2 and 3). Mothers with an Indigenous identity were less likely to use the formal family court system and more likely to have no formal custody arrangement compared to mothers with a White or Visible Minority background. In the absence of a formal custody arrangement, almost all the mothers reported having sole custody of their children. For those using the family court system, mothers and fathers with an Indigenous identity were less likely to be awarded joint custody but more likely for mothers to receive sole custody than mothers and fathers with a White or Visible Minority background. In the context of IPV, this may be interpreted positively as it may mean that Indigenous mothers were recognized for their important roles in protecting and nurturing their children as sole parents.

Regarding fathers, fewer fathers with reported Indigenous identity were awarded joint custody compared to fathers with reported White identity. As always, though, the experiences of Indigenous Canadians must be seen through the lenses of colonization and racism, especially as it negatively affects their interactions with the legal systems (Jock et al., 2022; Ogden & Tutty, 2023).

To the best of our knowledge, this is the first analysis of custody data in relation to ethnic background. Previous studies have shown how mothers with Indigenous identities are disadvantaged within the child welfare system where their parenting is scrutinized and problematized as deficient due to intergenerational trauma rather than the poverty and lack of educational opportunities and appropriate services frequently faced (e.g., Smye et al., 2021; Tutty & Nixon, 2020). The large percentage of women with Indigenous identities in our study who did not use the formal court system likely indicates concerns about how they will be viewed as mothers but is also consistent with Indigenous people's views of court involvement for other reasons (Jock et al., 2022).

In the current study, fathers had sole custody in a very small number of cases. The mother's problems with addictions and mental health issues were one reason for this, but some mothers also reported bias within the court system that disadvantaged them. It is notable that the mothers rarely willingly gave up responsibility for their children, and those who lost custody suffered profoundly from this loss. It is safe to say that the courts would do well to consider the steadfast nature of mothers’ commitments to their children even through difficult circumstances. Other research using the Healing Journey data examined mother's perspectives, competencies, and actions, concluding that most mothers with a history of IPV are “good” mothers and go to great lengths to protect and care for their children (e.g., Ateah et al., 2019; Nixon et al., 2017). On the other hand, the fathers in this study are not necessarily “good” fathers with mothers reporting a variety of negative effects on the children, including one child who was murdered by his father.

### Study Limitations and Strengths

When conducting secondary analyses, one is limited by the nature of the original study, which, in this case, relied on a convenience sample of women from VAW shelters or counseling agencies. Notably, most research on women and IPV does not involve a random selection of individuals from a population. Nevertheless, the current results may not be generalizable to other mothers abused by intimate partners from Canada's prairie provinces, particularly those who have not sought assistance for IPV.

As with most secondary analyses, the data were collected years before (2005–2009). It is not unusual to conduct secondary analyses on older data sets: As one example, Hilton et al. (2023) examined data from 1997. Although the findings must be considered through this lens, we argue for their relevance given the paucity of Canadian research on mother's perceptions of custody dispositions in the context of IPV, especially with Indigenous peoples.

Consistent with most research on IPV and custody decisions (an exception being Ogolsky et al., 2023), we did not have access to the actual custody dispositions, so relied on the women's self-reported custody arrangements. Had it been available when the study was conducted, the recently developed Legal Abuse Scale (Gutowski & Goodman, 2023), which documents coercive control used by partners with respect to the family court system, would have been invaluable.

The mother's comments were not gathered in in-depth interviews as was the case in other qualitative published studies on custody after IPV. Rather, they were in response to open-ended questions about custody arrangements among a longer set of questions on issues typically faced by women abused by intimate partners. Nevertheless, the comments provide a relatively complex, though brief view of what the women think of family court processes and outcomes.

Strengths of the current study include the quantitative analysis that adds to the almost exclusive qualitative nature of studies on child custody after IPV (exceptions being Birnbaum et al., 2017; Logan et al., 2003; Morrill et al., 2005). Further, the largest group of women are Indigenous, a group about which custody arrangements are missing in research. Importantly, in addition to family court dispositions, the analysis includes a little more than one-third of cases in which the partners did not engage with formal court processes, a novel research focus.

## Conclusion

Mothers with a history of IPV are typically concerned about their children's well-being and safety as paramount, and custody arrangements post-separation from a partner who has abused them have major consequences for them as well as their children (Tutty et al., 2025). This study has drawn attention to two virtually unstudied issues: (a) the large percentage of women with a history of IPV who have not pursued formal custody arrangements post-separation and (b) the experiences and perceptions of Indigenous mothers living in the prairie provinces who have separated from a partner who was abusive.

In relation to these two points, we argue that further changes to the justice system are required to offer a fair and supportive legal decision-making process for custody decisions in cases where IPV has occurred, and this is particularly the case for Indigenous mothers. The qualitative analysis of mothers’ perceptions of their child custody-related experiences confirms the conclusions drawn in qualitative studies with much smaller samples. Women and children who have lived with IPV continue to be at risk for an indeterminate amount of time post-separation. It is also clear that there is a lack of sufficient resources and support in these cases to ensure their safety and well-being in the future. We return to the values of the Duluth Model for inspiration on how to create legal interventions that better serve the needs of mothers and their children.
